# Nano Differential Scanning Fluorimetry-Based Thermal Stability Screening and Optimal Buffer Selection for Immunoglobulin G

**DOI:** 10.3390/ph15010029

**Published:** 2021-12-25

**Authors:** Soo Hyun Kim, Han Ju Yoo, Eun Ji Park, Dong Hee Na

**Affiliations:** 1 Department of Pharmacy, College of Pharmacy, Chung-Ang University, Seoul 06974, Korea; soohyun442@naver.com (S.H.K.); hanju2005@naver.com (H.J.Y.); 2 D&D Pharmatech, Seongnam 13486, Korea; ejpark@ddpharmatech.com

**Keywords:** nano differential scanning fluorimetry, immunoglobulin G, stability, aggregation, antibody formulation

## Abstract

Nano differential scanning fluorimetry (nanoDSF) is a high-throughput protein stability screening technique that simultaneously monitors protein unfolding and aggregation properties. The thermal stability of immunoglobulin G (IgG) was investigated in three different buffers (sodium acetate, sodium citrate, and sodium phosphate) ranging from pH 4 to 8. In all three buffers, the midpoint temperature of thermal unfolding (*T*_m_) showed a tendency to increase as the pH increased, but the aggregation propensity was different depending on the buffer species. The best stability against aggregation was obtained in the sodium acetate buffers below pH 4.6. On the other hand, IgG in the sodium citrate buffer had higher aggregation and viscosity than in the sodium acetate buffer at the same pH. Difference of aggregation between acetate and citrate buffers at the same pH could be explained by a protein–protein interaction study, performed with dynamic light scattering, which suggested that intermolecular interaction is attractive in citrate buffer but repulsive in acetate buffer. In conclusion, this study indicates that the sodium acetate buffer at pH 4.6 is suitable for IgG formulation, and the nanoDSF method is a powerful tool for thermal stability screening and optimal buffer selection in antibody formulations.

## 1. Introduction

Antibody drugs have become an important class after muromonab-CD3 (Orthoclone OKT3, Janssen-Cilag, Beerse, Belgium), the first antibody drug approved by the Food and Drug Administration in 1986 [1]. Since 1986, more than 100 antibody products have been approved in the United States or the European Union, and many candidates, including bispecific antibodies and antibody–drug conjugates, are in clinical trials [2,3,4,5]. With the recent rapid growth of antibody drugs, the development of formulations to ensure stability is becoming more important because antibodies are prone to various physical and chemical degradation processes, like any other proteins [6,7]. For successful formulation, several factors affecting the physicochemical stability of proteins must be considered and optimized, including solution pH, buffer species, and excipients [8].

Large and complex molecules, such as antibodies, are known to be prone to aggregation during the manufacturing processes and storage, which can lead to reduced potency and increased immunogenicity [9,10,11]. Aggregation is closely related to the thermal stability of antibody molecules. The lower the thermal stability, the less stable the product, and the higher degree of aggregation, whereas the higher thermal stability of the product can reduce the degree of aggregation [12]. Thermal stability analysis is traditionally performed using differential scanning calorimetry (DSC), but this technique is often limited to a certain concentration range and requires large amounts of sample (300–500 µL of samples per measurement) [13,14].

Differential scanning fluorimetry (DSF) is a fluorescence-based protein stability assay that measures protein folding state through monitoring changes in fluorescence as a function of temperature [15]. This technique provides biophysical properties such as midpoint temperature (*T*_m_) and onset temperature (*T*_onset_) of thermal unfolding. Conventional DSF is typically performed using a real-time polymerase chain reaction (PCR) instrument to monitor thermal denaturation of proteins in the presence of a fluorescent dye, such as SYPRO orange, that preferentially binds to unfolded proteins [16]. NanoDSF is a dye-free DSF method that monitors the change of intrinsic fluorescence from inherent tryptophan in protein as a function of temperature, time, or denaturant concentration [17]. Protein unfolding changes the microenvironment polarity around tryptophan residues, causing a red shift of fluorescence [18]; using this principle, nanoDSF determines *T*_m_ and *T*_onset_ by measuring the ratio of the fluorescence intensity at 330 nm and 350 nm as a function of temperature. The dye-free nature of nanoDSF makes it applicable to a wider range of protein samples than conventional DSF, which uses extrinsic dyes that can interact with surfactants often used in protein formulations [19]. In addition, nanoDSF offers the advantages of higher throughput and lower sample consumption than conventional approaches, such as DSC and circular dichroism [12]. The nanoDSF instrument used in this study (Prometheus NT.48, NanoTemper Technologies, München, Germany) can measure 48 samples in parallel within 90 min, requiring only 10 µL for each sample. In addition to measuring the intrinsic fluorescence intensity ratio (350/330 nm), this instrument uses backreflection technology to detect protein aggregation, providing the onset temperature of aggregation (*T*_agg_) with increasing temperature. Therefore, this instrument can simultaneously monitor protein unfolding transitions and aggregation.

In protein formulations, one of the important elements are buffers that control the pH of the formulation and can contribute to the stability of protein drug product by a variety of mechanisms [20]. In particular, the pH of the buffer is the most critical condition for protein formulation because pH itself has a greater effect on protein stability than any other factors [21,22]. The buffer species itself also affects the thermal stability of proteins beyond the direct effect of pH on stability [23]. One example is the difference in propensity for protein unfolding and aggregation between the sodium acetate and the sodium citrate buffers at the same pH as reported in stability studies with anti-streptavidin and anti-CD20 monoclonal antibodies [24,25,26]. Therefore, both pH and buffer species are key factors to consider when selecting the appropriate buffer for protein formulation.

In this study, a nanoDSF-based thermostability screening approach was applied to select the optimal buffer conditions for injectable liquid formulation of immunoglobulin G (IgG) as a typical model antibody [27,28]. The IgG used in this study was obtained from the I.V.-Globulin SN injection product from GC Pharma (Yongin, Korea), which is a pooled preparation of normal human IgG, obtained from healthy donors. It is widely used in the treatment of several autoimmune diseases and inflammatory bowel diseases, including Crohn’s disease and ulcerative colitis [29]. First, conformational stability and colloidal stability of IgG was investigated in three different buffers (sodium acetate, sodium citrate, and sodium phosphate), ranging from pH 4 to 8, using nanoDSF in order to select the optimal pH. Thereafter, thermal stability of IgG in the sodium acetate and sodium citrate buffers at the same pH was compared with their viscosity when highly concentrated over ~200 mg/mL. Finally, dynamic and static light scattering were performed to determine protein–protein interactions to elucidate stability mechanism in different buffers.

## 2. Results

### 2.1. NanoDSF Analysis of IgG

**Figure 1:**
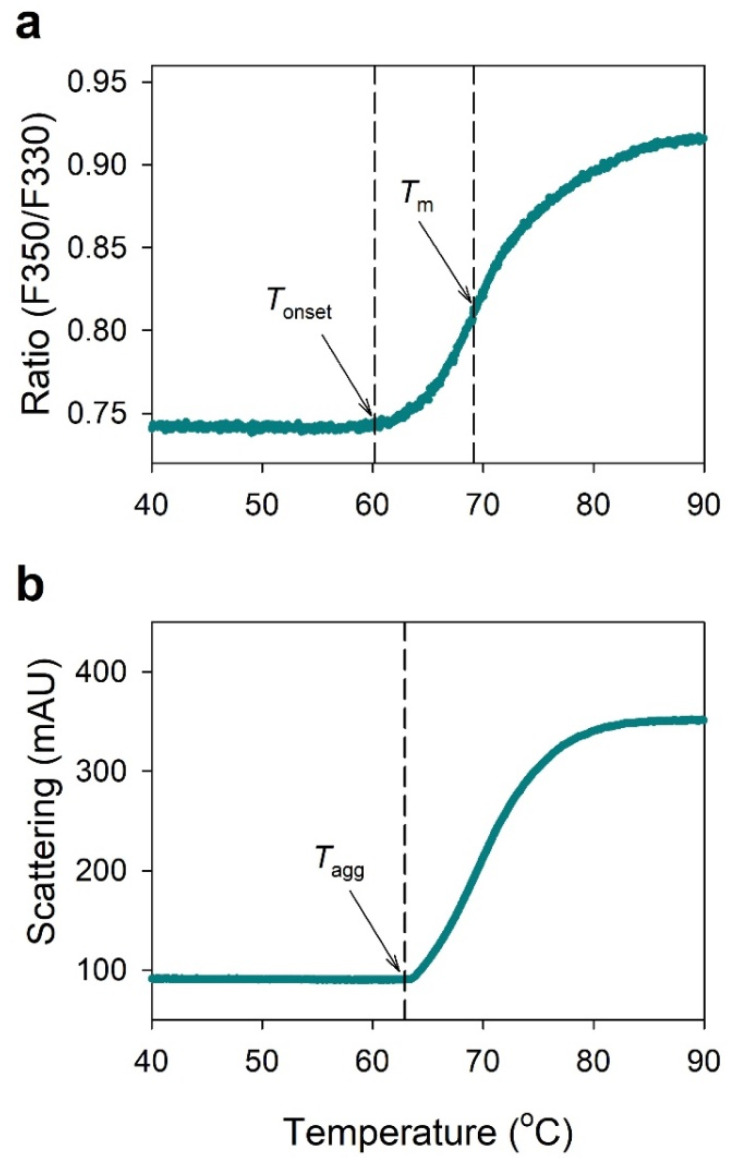
(**a**) Typical nanoDSF thermogram showing the fluorescence intensity ratio at 330 nm and 350 nm; (**b**) Light scattering thermogram by measuring the attenuation of the backreflected light intensity passing through the sample as a function of temperature. The sample was IgG at a concentration of 10 mg/mL in the 50 mM sodium phosphate buffer at pH 7.4. *T*_onset_—onset temperature of thermal unfolding; *T*_m_—inflection temperature of thermal unfolding; *T*_agg_—onset temperature of protein aggregation.

Figure 1 shows typical nanoDSF thermograms of IgG at a concentration of 10 mg/mL in the 50 mM sodium phosphate buffer at pH 7.4. Figure 1a shows the folding state transition of IgG by monitoring the ratio of fluorescence intensity at 330 nm and 350 nm as a function of temperature, where *T*_onset_ (onset temperature of thermal unfolding) was 60.2 °C and *T*_m_ (inflection temperature of thermal unfolding) was 69.2 °C. Figure 1b shows light scattering thermogram of IgG by measuring the attenuation of the backreflected light intensity passing through the sample, where *T*_agg_ (onset temperature at which a protein begins to aggregate) was 62.9 °C. This result shows the relationship between transition from folded state to unfolded state and aggregation of IgG.

**Figure 2:**
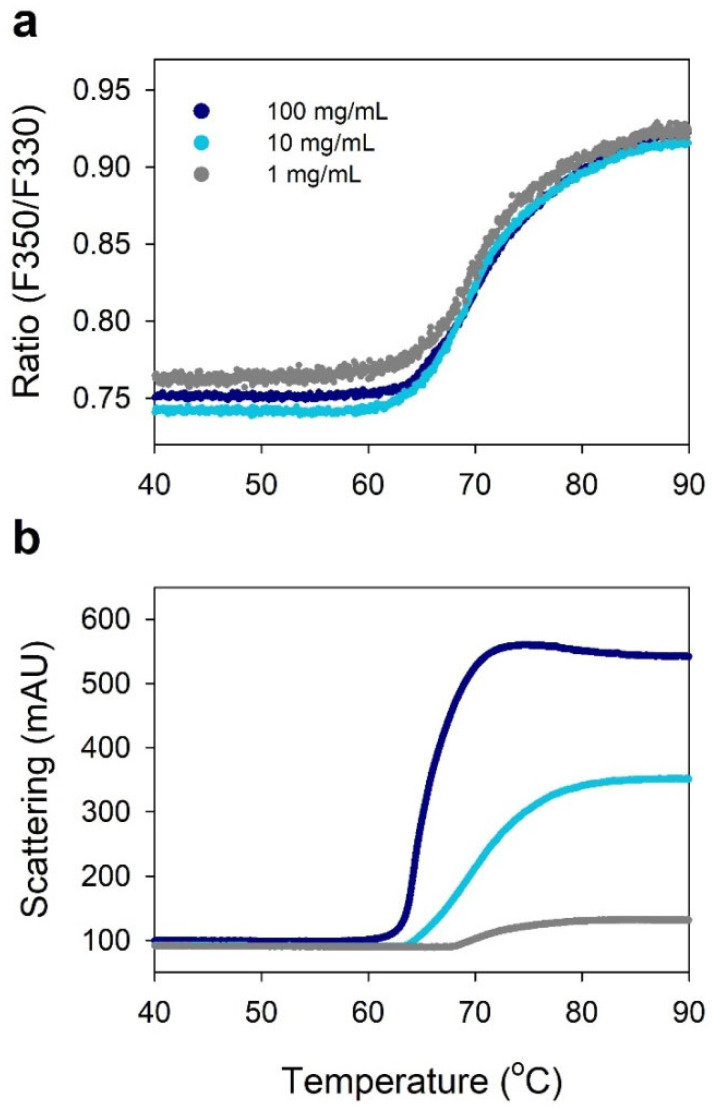
NanoDSF thermograms showing the effect of IgG concentration (gray—1 mg/mL; blue—10 mg/mL; dark blue—100 mg/mL) on the propensity for unfolding (**a**) and aggregation (**b**) of IgG in the 50 mM sodium phosphate buffer at pH 7.4. (**a**) NanoDSF thermogram; (**b**) Light scattering thermogram.

Figure 2 shows the effect of IgG concentration on the folding state transition and aggregation propensity of IgG. Different IgG concentrations of 1, 10, and 100 mg/mL in the 50 mM sodium phosphate buffer at pH 7.4 were tested. In this result, the effect of IgG concentration was not significant on the unfolding/denaturation of IgG, where *T*_onset_ results were 60.2 °C, 60.2 °C, and 61.4 °C, and *T*_agg_ results were 67.3 °C, 62.9 °C, and 56.6 °C, at IgG concentrations of 1, 10, and 100 mg/mL, respectively. However, the aggregation propensity was proportional to the concentration of IgG, where *T*_agg_ decreased and light scattering significantly increased as the concentration increased. This result suggests that both unfolding and aggregation should be considered for the development of highly concentrated antibody formulations. It also shows the utility of nanoDSF with backreflection technology that simultaneously monitors protein unfolding transitions and aggregation.

### 2.2. Buffer Screening by NanoDSF

**Figure 3:**
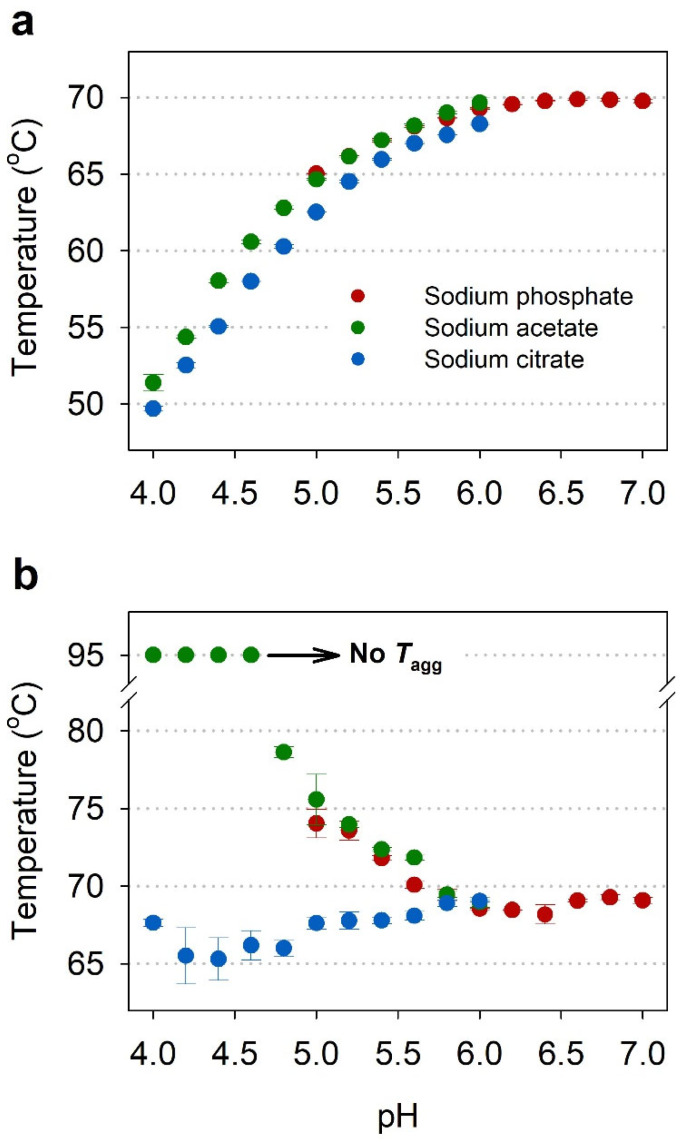
Effect of pH and buffer species on *T*_m_ (**a**) and *T*_agg_ (**b**) of IgG in three different buffers (red—sodium phosphate; green—sodium acetate; blue—sodium citrate), as measured by nanoDSF. (**a**) Graph of *T*_m_ of IgG as a function of pH; (**b**) Graph of *T*_agg_ of IgG as a function of pH. All samples were set at a concentration of 1 mg/mL and values are the mean and standard deviation of triplicate measurements.

The thermal stability of IgG was investigated in three different buffers (sodium acetate, sodium citrate, and sodium phosphate), ranging from pH 4 to 8, using nanoDSF in order to select the optimal pH (Table 1). The concentration of each sample was set to 1 mg/mL in order to reduce the sample consumption and conduct an efficient experiment. Figure 3a shows the profile of *T*_m_ as a function of pH for IgGs in different pH buffers, where higher *T*_m_ values were observed at higher pH. The *T*_onset_ values also showed the same trend as the *T*_m_ profile (Table 1). This means that conformational stability of IgG increases as the pH increases from 4 to 6 or higher.

Figure 3b shows the profile of *T*_agg_ as a function of pH for IgGs in different pH buffers. Unlike the *T*_m_ and *T*_onset_ results, the *T*_agg_ values were higher at acidic pH than neutral pH in the cases of the sodium acetate and phosphate buffers. In particular, in the acetate buffers at pH 4.6 and lower, aggregation did not occur even when heated to 95 °C. However, IgGs in the citrate buffers showed pH-independent aggregation with similar *T*_agg_ values of 65.3 to 69.0 °C. This result indicates that not only the pH but also the buffer species of the formulation affect the thermal stability of IgG.

### 2.3. Comparison of Thermal Stability between Acetate and Citrate Buffers

**Figure 4:**
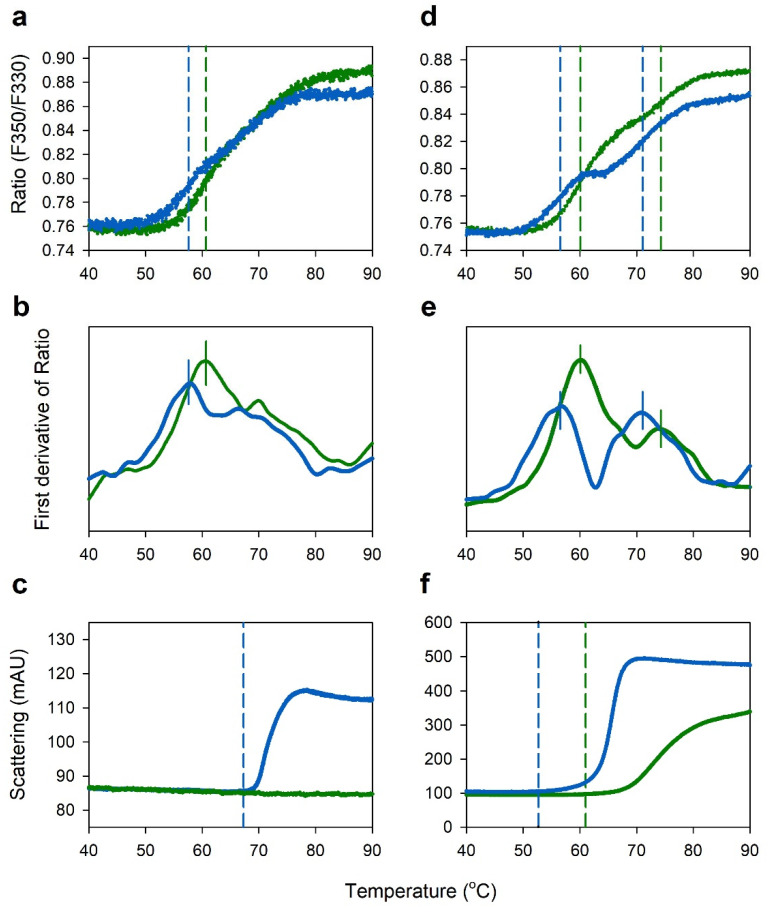
NanoDSF thermograms of IgGs at concentrations of 1 mg/mL (**a**–**c**) and 100 mg/mL (**d**–**f**) in acetate (green) and citrate buffers (blue) at the same pH 4.6. (**a,d)** fluorescence intensity ratio (F350 nm/F330 nm); (**b**,**e**) first derivative of fluorescence intensity ratio; (**c**,**f**) light scattering thermograms.

At pH 4.6, where no aggregation of IgG occurred in the sodium acetate buffer, as shown in Figure 3b, the effect of the sodium acetate and citrate buffers on thermal stability of IgG was studied by nanoDSF, with IgGs at concentrations of 1 and 100 mg/mL in 2 buffers at the same pH, 4.6. As shown in Figure 4a–c, 1 mg/mL IgG showed *T*_m_ values of 57.6 °C and 60.7 °C in the sodium citrate and acetate buffers, respectively, and *T*_agg_ of 66.7 °C in the sodium citrate buffer, but there was no aggregation in the sodium acetate buffer. Figure 4d–f shows thermograms of concentrated IgG at a concentration of 100 mg/mL. At this high concentration, two unfolding transitions (*T*_m1_ and *T*_m2_) were observed, where *T*_m1_ and *T*_m2_ are known to be attributed to unfolding transitions in the C_H_2 domain and the Fab/C_H_3 domain, respectively [12]. The *T*_m1_ and *T*_m2_ in the sodium citrate buffer were 56.5 °C and 71.1 °C, respectively, whereas those in the sodium acetate buffer were 60.1 °C and 74.3 °C, respectively. Unlike at 1 mg/mL, IgG at 100 mg/mL in the sodium acetate buffer also exhibited aggregation but showed higher *T*_agg_ of 61.0 °C and lower scattering intensity than *T*_agg_ of 52.7 °C in the sodium citrate buffer. Therefore, IgG showed higher thermal stability in the sodium acetate buffer with higher *T*_m_ and *T*_agg_ values than the sodium citrate buffer at the same pH, 4.6.

**Figure 5:**
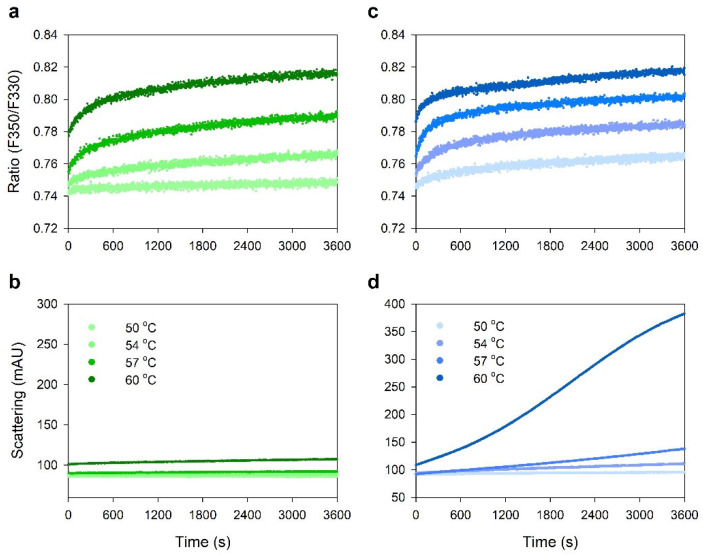
Isothermal stability of IgGs in acetate buffer (**a**,**b**) and citrate buffer (**c**,**d**) at the same pH of 4.6. (**a**,**c**) Fluorescence intensity ratio (F350 nm/F330 nm); (**b**,**d**) light scattering thermograms.

Figure 5 shows isothermal graphs of IgGs in the sodium acetate and sodium citrate buffers at pH 4.6. The fluorescence intensity ratio (F350/F330) and scattering property of the sample at each constant temperature of 50, 54, 57, and 60 °C were monitored for 60 min. As shown in Figure 5a,c, the fluorescence intensity ratio (F350/F330) of both IgGs in acetate and citrate buffers increased as the temperature increased, without significant difference between the two samples. However, there was significant difference of aggregation propensity between IgGs in acetate and citrate buffers at 60 °C, as shown in Figure 5b,d. This result also indicates that IgG is more stable in acetate buffer than in citrate buffer at the same pH of 4.6.

### 2.4. Viscosity of Highly Concentrated IgG

**Figure 6:**
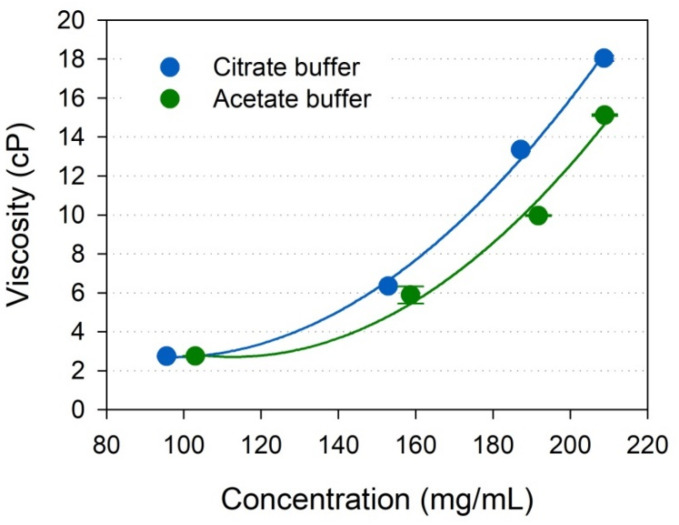
Viscosity profiles of IgG solutions from 95 mg/mL to over 209 mg/mL in acetate buffer (green) and citrate buffer (blue) at the same pH 4.6. Values are the mean and standard deviation from triplicate measurements.

The viscosity of IgGs in the sodium acetate and citrate buffers, that showed different thermal stability at the same pH, was measured by RheoSense microVISC viscometer employing rheometer-on-a-chip technology. Figure 6 shows the viscosity profiles of IgG solutions from 95 mg/mL to over 209 mg/mL in the sodium acetate and citrate buffers at the same pH of 4.6. In the sodium acetate buffer at pH 4.6, IgGs from 103.0 to 208.9 mg/mL exhibited viscosity from 2.75 ± 0.01 to 15.12 ± 0.05 cP, whereas in the sodium citrate buffer at the same pH, IgGs from 95.5 to 208.7 mg/mL showed viscosity from 2.74 ± 0.03 to 18.04 ± 0.15 cP. At relatively low concentrations around 100 mg/mL, IgGs in both buffers showed a similar viscosity of about 2.7 cP, but when concentrated to about 200 mg/mL, the IgG viscosity in the sodium acetate buffer was significantly lower than that in the sodium citrate buffer at the same pH, 4.6.

### 2.5. Protein–Protein Interactions

**Figure 7:**
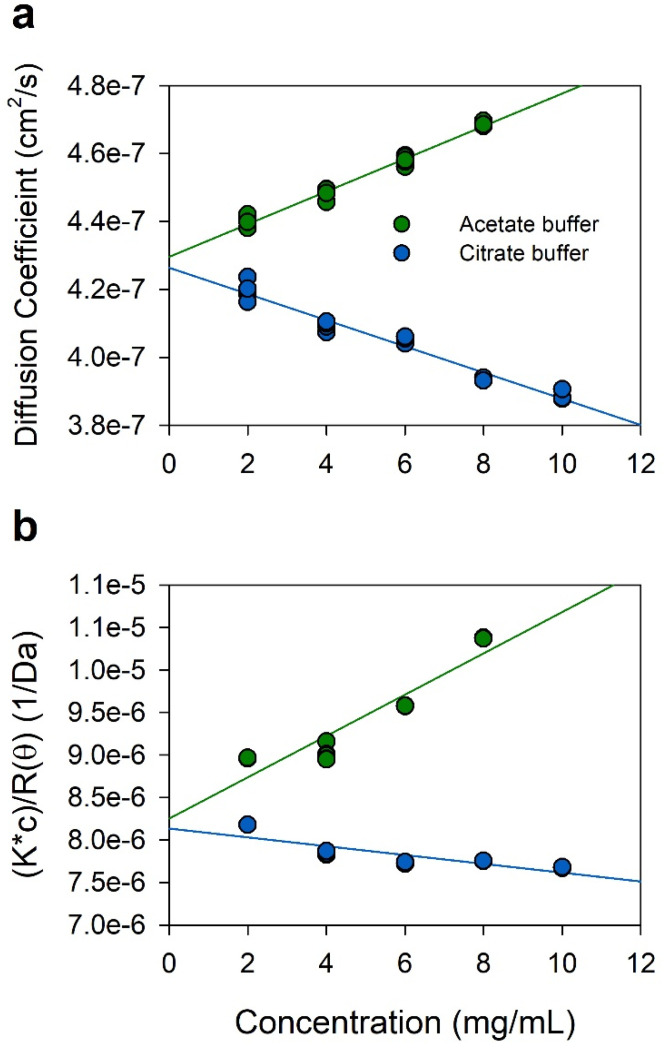
Protein–protein interactions in acetate buffer (green) and citrate buffer (blue) at pH 4.6. (**a**) The diffusion interaction parameter (*k*_D_) analysis by DLS; (**b**) the second virial coefficient (*A*_2_) measured by SLS. Values were obtained from quintuplicate measurements.

Protein–protein interactions (PPIs) are known to play a role as a major factor inducing aggregation and high viscosity in antibody formulations [30,31,32]. To elucidate the reasons for the difference in the degree of aggregation and viscosity of IgGs in the sodium acetate and citrate buffers, two measures of colloidal stability, the diffusion interaction parameter (*k*_D_) and the second virial coefficient (*A*_2_), were measured by DLS and SLS, respectively (Figure 7). Negative values of *k*_D_ and *A*_2_ mean molecular attraction and positive values mean molecular repulsion [33]. The *k*_D_ of IgG in the sodium acetate buffer was 11.2 mL/g, while the *k*_D_ of IgG in the sodium citrate buffer at the same pH 4.6 was −9.05 mL/g. Similar to *k*_D_, the *A*_2_ values of IgG in the sodium acetate and citrate buffers were 1.2145 × 10^−4^ and −2.5911 × 10^−5^ mol∙mL/g^2^, respectively. Consequently, intermolecular interactions were repulsive in the sodium acetate buffer at pH 4.6 but attractive in the sodium citrate buffer at pH 4.6. This result suggests that the differences between the sodium acetate and citrate buffers on the aggregation and viscosity of IgG correlate with protein–protein interactions.

## 3. Discussion

In this study, the nanoDSF method with high-throughput screening and aggregation measurement capabilities was applied to assess the conformational and colloidal stability of IgG at various pH conditions and to select the optimal buffer for IgG formulation. NanoDSF differs from conventional DSF in that it is a dye-free method that monitors changes in intrinsic fluorescence from the inherent tryptophan of a protein, as a function of temperature [15]. When the protein with tryptophan residues buried in the native state is exposed to strong thermal stress, the folded structure converts to an unfolded state and the tryptophan buried in the hydrophobic region is disclosed, thereby shifting the maximum emission wavelength from 330 nm to 350 nm (red shift) [18]. Figure 1a shows a typical nanoDSF thermogram of the F350/F330 ratio changes generated by the red shift of fluorescence due to the change in microenvironment polarity around the tryptophan residues. This demonstrates that IgG is suitable for nanoDSF measurement. On the other hand, for proteins with tryptophan residues exposed on the surface in the native state, unfolding simply causes a change in fluorescence emission intensity, but not at the F350/F330 ratio, which shows a flat signal in the nanoDSF thermogram [12]. In this case, application of nanoDSF is difficult.

NanoDSF determines *T*_m_ and *T*_onset_ by measuring the intrinsic fluorescence intensity ratio (350/330 nm) as a function of temperature, and its comparability with conventional DSF and DSC has been demonstrated in previous studies [9,12]. The nanoDSF instrument used in this study (Prometheus NT.48, NanoTemper Technologies) is equipped with backreflection technology that measures protein aggregation, providing the aggregation onset temperature (*T*_agg_) with increasing temperature. As shown in Figure 1, this instrument can simultaneously determine the conformational stability parameter, based on protein unfolding transitions (*T*_m_ and *T*_onset_), and the colloidal stability parameter, based on aggregation (*T*_agg_). This ability is important for determining the thermal stability of proteins as they may have different aggregation propensity despite similar *T*_m_ and *T*_onset_, as shown in Figure 2. The nanoDSF is a high-throughput screening method that can measure 48 samples in parallel within 90 min, requiring only 10 µL for each sample, which is useful for selecting optimal buffer conditions, as shown in Table 1 and Figure 3.

Human IgG consists of two identical light chains and two identical heavy chains linked together by disulfide bridges. Each heavy chain contains a variable domain (V_H_) and three conserved domains of C_H_1, C_H_2, and C_H_3, and each light chain contains a single variable domain (V_L_) and a single constant domain (C_L_). The V_H_ and C_H_1 domains form heterodimers with the light chain V_L_ and C_L_ domains corresponding to the Fab domain, and the C_H_2 and C_H_3 domains form the Fc fragment [34,35]. Previous work with DSC showed thermal unfolding curves of IgG presenting two distinctive endothermic peaks (*T*_m1_ and *T*_m2_), where *T*_m1_ peak was due to unfolding of the C_H_2 domain and *T*_m2_ peak was due to the unfolding of the Fab and C_H_3 domains [36]. The endothermic peaks were different depending on the pH, showing two broad peaks at pH 4 but and one large peak with a front shoulder at pH 6. A similar phenomenon was observed in this study as well, as shown in Figure 1, Figure 2, and Figure 4. Two unfolding transitions (*T*_m1_ and *T*_m2_) were observed under the acidic pH condition of 4.0–6.0, as shown in Figure 4d,e. However, as shown in Figure 1, only one *T*_m_ has observed at neutral conditions above pH 6, where the unfolding transition, due to the C_H_2 domain, disappeared, and the unfolding transition, due to the Fab and C_H_3 domains, were prominent.

The most important step to consider when developing liquid formulations of antibody drugs is the choice of buffer. In particular, the pH of the buffer is the most important condition for antibody formulation as shown in Figure 3, where pH itself had a greater effect on both the conformational and colloidal stability of IgG. Since the IgG used in this study is known to be basic with isoelectric points (pIs) of 6–9 [37,38], it was expected that acidic buffers lower than pH 6 would exhibit stability against aggregation. Moreover, many immunoglobulin products have been formulated at acidic pH [39]. As acidic buffers, acetate and citrate buffers are most commonly used for antibody formulations [40]. On the other hand, commercially available antibody formulations have a pH range of up to 8.0 [40]. Therefore, a phosphate buffer was used to cover the neutral pH range. In addition to buffers, additives for antibody formulation may include tonicity-adjusting excipients, viscosity-lowering excipients, and surfactants [40]. Sodium chloride is mainly used as an ionic tonicity-adjusting excipient. Sugars and polyols, such as sucrose, trehalose, mannitol, maltose, and sorbitol, are used as non-ionic osmolality-adjusting excipients. Surfactants are included in antibody formulation to inhibit aggregation and minimize surface adsorption at the air–water interface and containers. The most common surfactants are polysorbate 20 and polysorbate 80 [41]. If these additives are added to the formulation, the formulation with higher stability may be obtained.

The effects on the thermal stability of IgG in the sodium citrate buffers were different from the sodium acetate buffers at the same pH. As shown in Figure 4, the IgG in the sodium citrate buffer exhibited lower *T*_m_ and *T*_agg_ values than those of IgG in the sodium acetate buffer at the same pH 4.6. In isothermal stability at 60 °C, IgG was also more stable in the sodium acetate buffer than in the sodium citrate buffer, as shown in Figure 5. Differences in unfolding and aggregation propensities between the sodium acetate and citrate buffers at the same pH have also been reported in stability studies with anti-streptavidin IgG1 and anti-CD20 monoclonal antibody Mab-T [24,25,26]. Barnett et al. reported that anti-streptavidin IgG1 exhibited higher *T*_m_ and stronger net repulsive PPIs in acetate buffer compared with the citrate buffer [25]. They speculated that preferential accumulation of citrate anions on the protein surface compared with acetate anions would reduce electrostatic repulsions between antibodies. Oyama et al. also reported similar buffer effect on Mab-T antibody stability, showing higher *T*_agg_ and repulsive PPIs between antibodies in acetate buffer at pH 5.0 compared with the citrate buffer at the same pH 5.0 [26]. They also suggested that the charge-shielding effect of citrate anions accumulated on the surface of the Mab-T antibody is a major source of lower colloidal and conformational stability in the sodium citrate buffer. In this study, the *k*_D_ and *A*_2_ values of IgG in acetate buffer was positive, whereas the values of IgG in citrate buffer at the same pH 4.6 was negative, indicating that intermolecular interactions were repulsive in acetate buffer at pH 4.6 but attractive in citrate buffer at pH 4.6 (Figure 7). This result is in good agreement with the aforementioned studies of anti-streptavidin IgG1 and Mab-T antibody [24,25,26]. Contrasting interactions in the two buffers resulted in differences in viscosity when IgG was concentrated to high concentrations as shown in Figure 6. When concentrated to about 209 mg/mL in each buffer, the IgG viscosity was 15.12 ± 0.05 cP in the sodium acetate buffer, vs. 18.04 ± 0.15 cP in the sodium citrate buffer at the same pH 4.6. Therefore, *k*_D_ and *A*_2_ values showed a good correlation to the aggregation and viscosity propensity of IgG.

High protein concentrations above 50 mg/mL are becoming increasingly important for antibody formulations. High antibody concentrations are also essential for intravenous or subcutaneous IgG administration for a replacement therapy of primary immunodeficiency diseases and for modulation of various autoimmune or inflammatory diseases [42,43]. In particular, in the case of subcutaneous administration, since the maximum volume is limited to 1.0–1.5 mL, a highly concentrated protein composition is required to administer a high-dose therapeutic antibody [44]. However, highly concentrated antibody solutions greatly increase the viscosity, complicating patient administration by subcutaneous injection [45]. Therefore, viscosity is a critical quality attribute of highly concentrated antibody formulations. In this study, when concentrated to approximately 209 mg/mL in the sodium acetate buffer at pH 4.6, the viscosity of the IgG solutions was 15.12 ± 0.05 cP (Figure 6), which is comparable to the viscosity of 14.7 ± 1.2 cP of the commercial product IgPro20 (Hizentra^®^, CSL Behring, Berne, Switzerland), a 20% liquid preparation of IgG for subcutaneous administration [46].

## 4. Materials and Methods

### 4.1. Materials

Human IgG was obtained from I.V.-Globulin SN injection product from GC Pharma (Yongin, Korea). Acetic acid glacial was purchased from Duksan (Ansan, Korea). Tergazyme was purchased from Alconox (White Plains, NY, USA). All other reagents including buffer reagents and chemicals, unless indicated otherwise, were obtained from Sigma-Aldrich (St. Louis, MO, USA).

### 4.2. Buffer Preparation

Three different types of buffers (sodium phosphate, sodium acetate, and sodium citrate) were prepared using deionized water (18.2 MΩ∙cm; Millipore, Billerica, MA, USA). The pH of the buffer was measured with a pH meter (SevenCompact pH meter S220, Mettler Toledo AG, Schwerzenbach, Switzerland) and adjusted to the target value (within ± 0.05 deviation). The buffer concentration was adjusted to the same concentration as 50 mM. The prepared buffers were filtered through 0.20 µm cellulose acetate filter (Toyo Roshi Kaisha Ltd., Tokyo, Japan).

### 4.3. Buffer Exchange and Centrifugal Concentration

IgG with a protein concentration of 50 mg/mL was originally formulated in the product with a 100 mg/mL maltose solution at pH 4.25. In order to remove excipients in the product and formulate the IgG with the target buffer, the product solution was first exchanged with a low ionic strength buffer (5 mM sodium phosphate buffer at pH 5.5) using an ultrafiltration unit with molecular weight cut-off (MWCO) of 30 kDa (Vivaspin Turbo 15 RC; Sartorius AG, Göttingen, Germany) at a spin speed of 3000 rcf for 15 min. This was performed at 4 °C by mixing the product solution and the 5 mM sodium phosphate buffer (pH 5.5) at a ratio of 1:1 and concentrating to the original concentration and repeated 6 times. After this, the solutions were diluted with each target buffer to protein concentrations of 1, 2, 10, and 100, or greater than 200 mg/mL for nanoDSF experiments. Protein concentration was examined by UV absorbance at 280 nm using Lunatic (Unchained Labs, Pleasanton, CA, USA), which directly measures a wide concentration range of protein solutions without dilution and required only a volume of 2 µL.

### 4.4. Nano Differential Scanning Fluorimetry

NanoDSF was performed using Prometheus NT.48 equipped with backreflection mode (NanoTemper Technologies, München, Germany). Samples were loaded in nanoDSF grade standard capillaries (NanoTemper Technologies GmbH, München, Germany) and exposed at thermal stress from 20 °C to 95 °C by thermal ramping rate of 1 °C/min. Fluorescence emission from tryptophan after UV excitation at 280 nm was collected at 330 nm and 350 nm with dual-UV detector. Protein aggregation was assessed simultaneously employing backreflection optics, which detects protein aggregation by measuring the attenuation of backreflected light intensity passing through the sample. Thermal stability parameters, including *T*_onset_, *T*_m_, and *T*_agg_, were calculated by PR.ThermControl software (NanoTemper Technologies, München, Germany). For isothermal stability, the time interval data from thermal stress at constant temperature (50, 54, 57, or 60 °C) were collected by PR.TimeControl software (NanoTemper Technologies, München, Germany).

### 4.5. Viscosity Measurement

Viscosity of samples was measured by microVISC viscometer (RheoSense, San Ramon, CA, USA) employing Rheometer-on-a-chip technology. A05 chip with viscosity ragne of 0–100 mPa∙s (cP) was used. Before measurements, the chip was thoroughly cleaned with tergazyme solution and calibrated with Newtonian fluids standard (water). Approximately 400 µL of the IgG samples was carefully loaded into a disposable syringe and the syringe was placed in a viscometer, maintained at a temperature of 25 ± 0.1 °C in a temperature control unit (HVROC-T). After temperature equilibration for 5 min, samples were injected through a 50 µm flow channel at a constant flow rate, selected automatically.

### 4.6. Dynamic and Static Light Scattering

In order to measure the solute–solute and solute–solvent interactions, diffusion interaction parameter (*k*_D_) and the second virial coefficient (*A*_2_) were determined using DynaPro NanoStar (Wyatt Technology Corporation, Santa Barbara, CA, USA), which can simultaneously perform dynamic light scattering (DLS) and static light scattering (SLS) with a laser wavelength of 661 nm. Samples of IgG formulations ranging from 2 to 10 mg/mL were applied. By DLS, the diffusion coefficient was plotted as a function of sample concentration; thus, *k*_D_ was calculated according to Equation (1), as follows:

*D*_m_ = *D*_0_ (1 + *k*_D_ c) (1)

where *D*_m_ is the measured diffusion coefficient, *D*_0_ is the diffusion coefficient at infinite dilution, and *c* is the concentration of protein (mg/mL).

*A*_2_ value was calculated based on the slope of static light scattering at 90° as a function of concentration, according to Equation (2), as follows:

(2) $$\frac{K\times c}{R\left(\theta \right)}=\frac{1}{M}+2A_{2}c$$

where *K* is optical constant, *c* is concentration of protein, *M* is apparent molar mass, and R(θ) is the excess Rayleigh ratio.

## 5. Conclusions

This study demonstrates the utility of a nanoDSF-based thermostability assessment technology for the development of IgG formulations. The nanoDSF method could simultaneously monitor conformational and colloidal stability of IgG. As a result of monitoring in three different buffers (sodium acetate, sodium citrate, and sodium phosphate) in the pH 4–8 range, the best stability against aggregation was obtained in the sodium acetate buffers at pH 4.6 or lower. On the other hand, IgG in the sodium citrate buffers showed higher aggregation and viscosity than in the sodium acetate buffers at the same pHs. The PPI parameters measured by DLS and SLS indicated that intermolecular interactions were repulsive in acetate buffer at pH 4.6 but attractive in citrate buffer at pH 4.6. These results suggest that the binding of citrate anions to the surface of IgG leads to attractive PPIs, and has a lower *T*_agg_ than acetate buffers, in which repulsive PPIs occur. In conclusion, the sodium acetate buffer at pH 4.6 is recommended as a buffer for IgG formulation, and the nanoDSF method would be useful for the formulation and development of various protein drugs, as well as other antibodies.

## Figures and Tables

**Table 1:** Thermal stability of IgGs in three buffers with different pHs as measured by nanoDSF.

Buffers	pH	Thermal Stability Parameters (°C)		
		*T*_onset_	*T*_m_	*T*_agg_
Sodium phosphate	5.0	57.9 ± 0.5	65.0 ± 0.0	74.0 ± 0.9
	5.2	58.7 ± 0.8	66.1 ± 0.0	73.6 ± 0.6
	5.4	59.9 ± 0.4	67.2 ± 0.1	71.8 ± 0.2
	5.6	60.7 ± 0.9	68.1 ± 0.0	70.1 ± 0.2
	5.8	61.2 ± 0.3	68.7 ± 0.0	69.4 ± 0.4
	6.0	61.4 ± 0.1	69.3 ± 0.1	68.5 ± 0.0
	6.2	61.6 ± 0.3	69.6 ± 0.0	68.5 ± 0.0
	6.4	61.8 ± 0.4	69.8 ± 0.0	68.2 ± 0.6
	6.6	61.4 ± 0.0	69.9 ± 0.1	69.1 ± 0.1
	6.8	60.1 ± 0.3	69.8 ± 0.1	69.3 ± 0.2
	7.0	60.8 ± 0.1	69.8 ± 0.1	69.1 ± 0.2
	7.3	60.4 ± 0.5	69.5 ± 0.1	69.5 ± 0.2
	7.5	60.2 ± 0.2	69.4 ± 0.1	70.1 ± 0.1
	7.7	59.7 ± 0.9	69.2 ± 0.0	69.2 ± 0.6
	8.0	58.9 ± 0.2	69.0 ± 0.0	70.5 ± 0.5
Sodium acetate	4.0	42.3 ± 0.1	51.4 ± 0.5	No aggregation
	4.2	45.0 ± 0.0	54.4 ± 0.1	No aggregation
	4.4	50.1 ± 0.5	58.0 ± 0.1	No aggregation
	4.6	53.2 ± 0.1	60.6 ± 0.1	No aggregation
	4.8	55.2 ± 0.2	62.8 ± 0.1	78.6 ± 0.4
	5.0	57.0 ± 0.3	64.6 ± 0.1	75.6 ± 1.6
	5.2	58.6 ± 0.1	66.1 ± 0.0	74.0 ± 0.2
	5.4	59.9 ± 0.1	67.2 ± 0.0	72.4 ± 0.1
	5.6	60.3 ± 0.5	68.2 ± 0.1	71.8 ± 0.2
	5.8	60.9 ± 0.3	69.0 ± 0.1	69.5 ± 0.2
	6.0	61.4 ± 0.3	69.7 ± 0.4	69.0 ± 0.3
Sodium citrate	4.0	40.1 ± 0.2	49.7 ± 0.2	67.6 ± 0.2
	4.2	44.3 ± 0.1	52.5 ± 0.2	65.5 ± 1.8
	4.4	47.6 ± 0.3	55.1 ± 0.1	65.3 ± 1.4
	4.6	50.5 ± 0.5	58.0 ± 0.1	66.2 ± 0.9
	4.8	53.0 ± 0.5	60.3 ± 0.1	66.0 ± 0.5
	5.0	55.4 ± 0.1	62.5 ± 0.0	67.6 ± 0.4
	5.2	57.7 ± 0.0	64.5 ± 0.1	67.8 ± 0.5
	5.4	58.4 ± 0.4	66.0 ± 0.1	67.8 ± 0.2
	5.6	59.7 ± 0.3	67.0 ± 0.0	68.1 ± 0.3
	5.8	60.2 ± 0.5	67.6 ± 0.0	68.9 ± 0.2
	6.0	60.6 ± 0.2	68.3 ± 0.0	69.0 ± 0.1

Values are the mean and standard deviation from triplicate measurements.

## Data Availability

Data is contained within the article.
